# Utilizing allogeneic venous grafts to solve the problem of vascular defects and reconstruction in pancreatic surgery: a retrospective cohort study

**DOI:** 10.3389/fmed.2026.1875288

**Published:** 2026-08-26

**Authors:** Lin Zhou, Shao-cheng Lyu, Han-xuan Wang, Hua Fan, Tao Jiang, Ren Lang, Qiang He

**Affiliations:** Division of Hepatobiliary and Pancreaticosplenic Surgery, Department of General Surgery, Beijing Chao-Yang Hospital, Capital Medical University, Beijing, China

**Keywords:** postoperative prognosis, stenosis, surgical technique, vascular graft, venous invasion

## Abstract

**Background:**

Extensive vascular invasion or defects after resection significantly limit the feasibility of radical pancreatic surgery, underscoring the value of effective vascular reconstruction techniques. This study evaluates a novel approach to vascular reconstruction using allogeneic venous grafts (AVGs) in pancreaticoduodenectomy and pancreatectomy.

**Methods:**

This retrospective cohort study included 187 patients with biliary or pancreatic malignancies or lesions presenting with various types of venous invasion who underwent pancreatic resection with vascular reconstruction using AVGs. The R0 resection rate, long-term patency rate of reconstructed vessels, and overall survival (OS) outcomes were evaluated. Univariate and multivariate Cox regression and logistic regression analyses were performed to identify independent risk factors for postoperative vascular stenosis and poor survival.

**Results:**

All patients presented with variable extents of vascular defects and anatomical sites of vascular invasion, including the portal vein–superior mesenteric vein confluence (*n* = 110), superior mesenteric vein (*n* = 50), portal vein (*n* = 25), inferior vena cava (*n* = 1), and left renal vein (*n* = 1). The postoperative complication rate was 33.2%, and the perioperative mortality rate was 7.0% both of which were comparable across different AVG reconstruction configurations. Excluding benign lesions, the overall R0 resection rate was 92.2% (166/180) across the malignant cohort, specifically 92.3% (132/143) in patients with pancreatic cancer, and 90.0% (27/30) in those with cholangiocarcinoma. The median follow-up duration was 88.0 months. Cumulative graft stenosis rates at 1-year, 2-year, and 3-year postoperatively were 18.4, 21.8, and 33.3%, respectively. Median overall survival and disease-free survival were 13 and 9 months, respectively, with 5-year overall survival and disease-free survival rates of 12.2 and 12.7%. The type of AVG used for vascular reconstruction had no significant short-term or long-term oncological outcomes (both *p* > 0.05).

**Conclusion:**

The use of AVGs is a feasible and effective technique for vascular reconstruction following major venous resection in patients with locally advanced pancreatic and biliary malignancies. Its application achieved favorable R0 resection rates and acceptable long-term patency and survival outcomes.

## 1 Introduction

Vascular invasion and secondary defects are critical determinants of surgical resectability and technical success in complex abdominal surgery, and allogeneic venous vessel grafts (AVGs) represent a promising conduit choice to address this challenge. AVGs were first introduced for portal vein (PV) and inferior vena cava (IVC) reconstruction in liver transplantation several decades ago (1–3). With advancements in microvascular techniques and organ preservation, AVGs have since been widely applied across a broad range of abdominal oncological and transplant procedures (2–8).

In hepatobiliary, pancreatic, urological, and retroperitoneal oncology, tumor invasion into adjacent major abdominal vessels frequently necessitates vascular resection (8–10). In addition, vascular reconstruction is required in liver and kidney transplantation, retroperitoneal vascular leiomyosarcoma, and aortic-related diseases (2, 3, 6, 7, 11). Failure to reconstruct a vascular defect or restore the continuity of an important vascular axis often precludes R0 radical resection, highlighting the necessity of established vascular reconstruction techniques and an optimal vascular substitute (12). Currently, four principal conduit categories are employed for SMV–PV reconstruction: autologous venous grafts, autologous non-vascular tissue grafts, synthetic grafts, and AVGs (13). Although autologous venous grafts offer excellent biocompatibility, their harvest introduces donor-site morbidity, including lower extremity wound complications and chronic edema, prolongs operative time, and frequently presents a size-mismatch between the harvested vein and the recipient PV/SMV (14). Non-vascular tissue grafts, such as the falciform ligament, are largely limited to patch repair rather than circumferential segmental replacement and carry a high incidence of graft thrombosis (15). Synthetic PTFE grafts, while offering immediate off-the-shelf availability, carry the highest thrombosis rates among all conduit types, often requiring long-term or lifelong anticoagulation. Furthermore, the use of prosthetic materials is generally contraindicated in the inherently contaminated operative field of pancreatic resection due to the risk of graft infection and secondary blowout hemorrhage (13). In contrast, AVGs offer numerous clinical advantages, including of abundant sources, optional vascular diameter and length, excellent plasticity, resistance to infection, good tissue compatibility, conformity with physiological characteristics, unnecessity of routine anticoagulants, and avoidance of prolonged operation time and increased trauma caused by autologous blood vessels harvest (4, 16). Besides, AVGs also enable the reconstruction of multi-branch vessels and can achieve tension-free anastomosis with good compatibility during vascular reconstruction, reducing the risk of serious postoperative complications. Due to the above-mentioned advantages, AVGs have gained increasing adoption in hepatopancreatobiliary surgery.

Currently, AVGs have been widely adopted and integrated into routine surgical management for pancreatic cancer with vascular invasion at our center. Based on a cohort of 298 patients presenting with locally advanced pancreatic cancer and vascular invasion, we previously proposed the Chaoyang classification of vascular invasion, which categorizes PV–superior mesenteric vein (SMV) invasion into four types based on operative complexity and extent of venous involvement (5). Briefly, Type I denotes tumor abutment involving less than one-quarter of the vessel wall circumference, managed by partial venous excision with direct venorrhaphy. Type II denotes tumor abutment involving more than one-quarter of the circumference of SMV or PV circumference, managed by segmental resection with either direct end-to-end venous anastomosis or an interposed AVG reconstruction. Type III denotes tumor encasement of the PV–SMV confluence, requiring segmental resection with AVG graft in all cases. Type IV describes tumor encasement involving the PV–SMV confluence and extending into the main PV trunk and first-order SMV branches, requiring the most extensive multibranch reconstruction using AVG interposition (17). The implementation of AVGs significantly improved the R0 resection rate and overall survival of patients with pancreatic cancer at our center, particularly in those with types III–IV vascular invasion under the Chaoyang classification, demonstrating the feasibility of AVG reconstruction in pancreatic resection. Subsequently, we extended the application of this technique to the radical surgical resection of other hepatobiliary and pancreatic malignancies with vascular invasion, significantly expanding the surgical indications for these complex tumors. Thus, we hypothesized that the application of AVGs is a safe and effective method of vascular reconstruction in pancreatic surgery, yielding high R0 resection rates and acceptable long-term vascular patency rates. The present study aims to summarize the application patterns and surgical scenarios of AVGs in pancreatic surgery based on our institutional experience and to evaluate their post-operative and oncological outcomes, with the goal of informing a standardized treatment protocol for AVG-based vascular reconstruction.

## 2 Methods

### 2.1 Patient selection

This was a single-center, retrospective observational cohort study. Patients with suspected or confirmed biliary and pancreatic lesions with suspected invasion into important major visceral veins who were admitted to Beijing Chao-Yang Hospital, Capital Medical University between January 1, 2013, and June 30, 2024, were screened for eligibility based on predefined inclusion and exclusion criteria. The study protocol was approved by the Ethics Committee of Beijing Chao-Yang Hospital, Capital Medical University. The requirement for written informed consent was waived by the Ethics Committee of Beijing Chao-Yang Hospital, Capital Medical University due to the retrospective nature of the study (granted no. 2023-D-437). This study was conducted and reported in accordance with the Strengthening the Reporting of Observational Studies in Epidemiology (STROBE) guidelines (18).

### 2.2 Inclusion and exclusion criteria

#### 2.2.1 Inclusion criteria

(1) Preoperative imaging demonstrating a hepatobiliary or pancreatic tumor with suspected invasion into major abdominal veins; (2) Preoperative or intraoperative assessment confirming the necessity of vascular resection due to tumor invasion or extensive adhesion; (3) Receipt of R0 or R1 radical en-bloc resection of the tumor and involved vessels; (4) Utilization of AVGs for vascular reconstruction.

#### 2.2.2 Exclusion criteria

(1) Preoperative imaging demonstrating distant metastasis or other surgical contraindications; (2) Absence of vascular invasion or tumor involvement during intraoperative exploration; (3) Unresectable disease identified during intraoperative evaluation; (4) Vascular reconstruction achieved via primary end-to-end anastomosis without graft interposition; (5) Incomplete clinical records or loss to follow-up.

**Figure 1:**
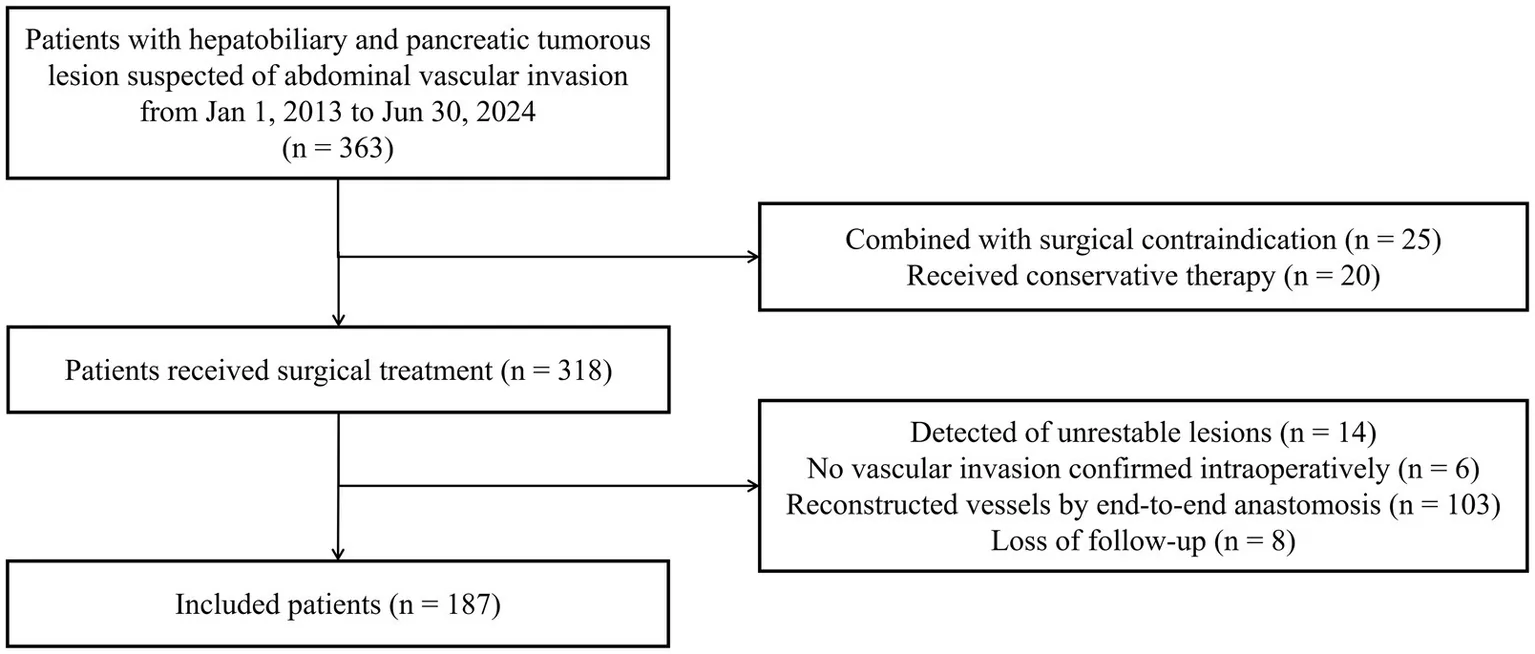
Flow diagram of patients selection.

The flow chart of patient selection is presented in Figure 1.

### 2.3 Protocol of diagnosis and treatment

Upon admission, all enrolled patients underwent comprehensive preoperative laboratory testing, including complete blood count, serum biochemistry, serum tumor markers, and coagulation profiles. Contrast-enhanced abdominal computed tomography (CT) or magnetic resonance imaging (MRI) were routinely performed. CT angiography (CTA) with three-dimensional vascular reconstruction was performed when vascular invasion was suspected; positron emission tomography-computed tomography (PET–CT) or bone scintigraphy were utilized to exclude distant metastasis. Patients without distant metastases or technical/medical contraindications underwent radical tumor resection with en-bloc vascular resection and AVG under general anesthesia.

### 2.4 Acquisition, preservation, and selection of AVGs

AVGs utilized during surgery were harvested from iliac veins procured from brain-dead organ donors. The procurement protocol was approved by the ethics committee of our hospital, and written informed consent was obtained from the donors’ families. The harvested AVGs were trimmed under sterile conditions by a surgeon after acquisition, during which the surrounding adipose tissue was removed, and small vascular branches were ligated. The prepared AVGs were then preserved in sterile physiologic saline at 4 °C. The donor organ preservation solution and the normal saline used for vessel storage underwent etiological examination before application.

ABO blood group compatibility and preservation duration were strictly evaluated for AVG selection before surgery. Only AVGs from ABO-identified or compatible donors were selected for reconstruction. AVGs preserved for less than 7 days were preferentially selected. AVGs preserved for 7–14 days were used only if no fresher AVG was available. AVGs preserved for longer than 14 days were discarded from clinical reconstruction. Before implantation, the selected AVG was rewarmed to room temperature and thoroughly flushed with heparinized saline before clinical utilization.

### 2.5 Protocol of surgery and vascular reconstruction

**Figure 2:**
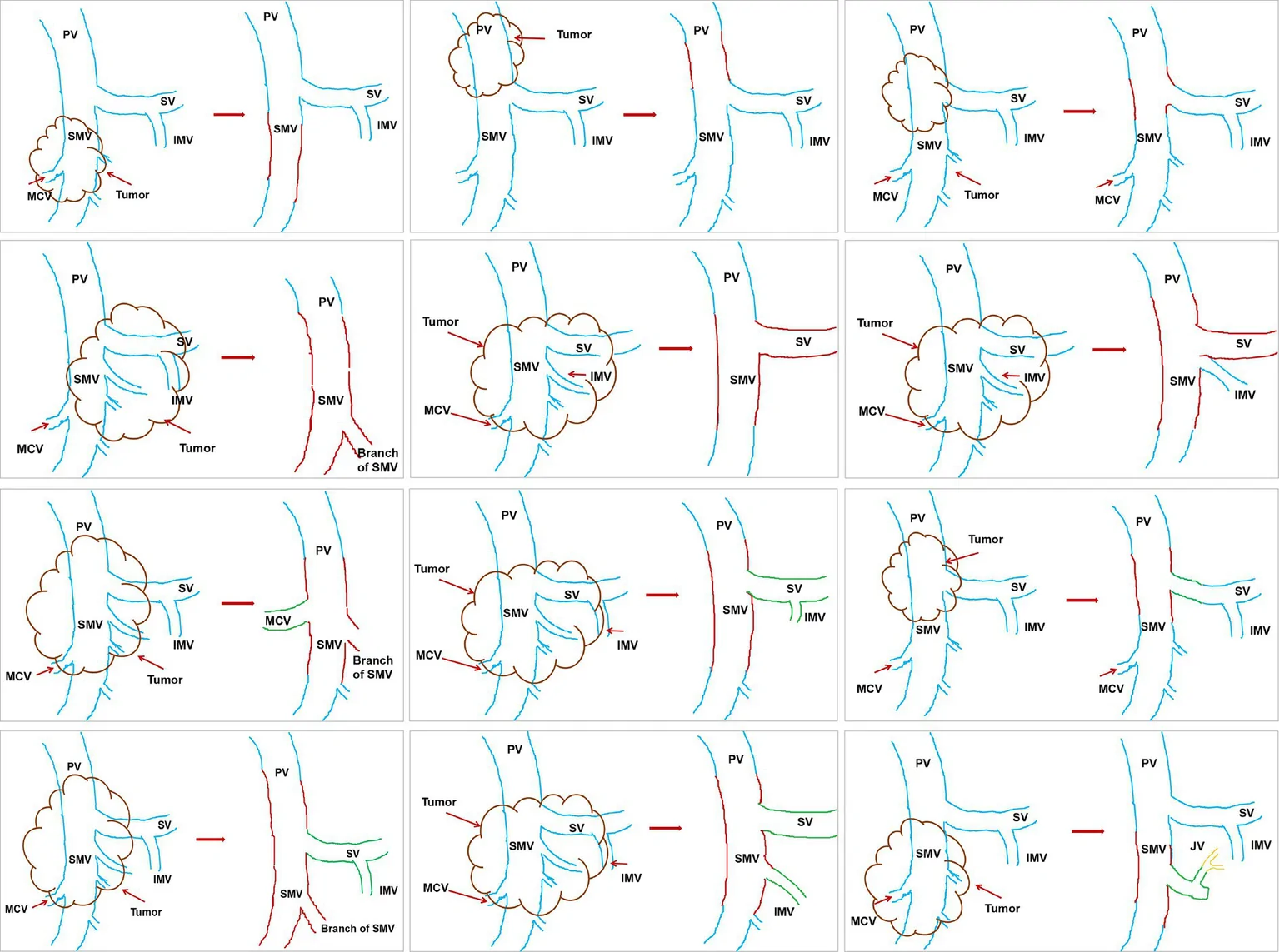
Protocol of vascular reconstruction under different vascular invasion situations: Tubular (I-shaped) or branched (Y-shape, L-shape, and T-shape) AVGs are applied in reconstructing tubular, branched, or multibranched vascular defects after resection. AVG, Allogeneic venous graft; Vessels marked with non-blue color refer to AGVs.

During the preoperative counseling, all patients eligible for AVG reconstruction were fully informed of the source, clinical necessity, expected outcomes, and potential risks of AVG use, and signed a specific informed consent form detailing all of the aforementioned information. All surgical procedures were guided by intraoperative exploration, and all included patients underwent curative-intent surgery with systemic lymph node dissection. To achieve true en-bloc resection in patients with tumor-related vascular invasion, a tailor-made vascular reconstruction protocol utilizing AVGs was implemented to accommodate varying extents of vascular invasion and resultant wall defects. Straight tubular grafts, single-branch grafts, and composite multi-graft constructs were utilized to re-establish portal-mesenteric venous continuity, ensuring unobstructed visceral venous drainage, as shown in Figure 2.

### 2.6 Postoperative management and follow-up plan

Patients who underwent vascular reconstruction with AVGs were administered subcutaneously injected low-molecular weight heparin calcium (0.4 mL/day) from postoperative day (POD) 2 to POD 7, followed by oral aspirin (100 mg/day) or rivaroxaban (10–15 mg/day) for 6 months. Color duplex ultrasonography was routinely performed on POD 3 and POD 7 to evaluate flow dynamics and patency within the reconstructed vessels. Abdominal-enhanced CT was routinely arranged at POD 30 to assess long-term vascular graft patency.

Postoperative follow-up was scheduled every 3 months during the first 2 years and every 6 months thereafter to evaluate clinical status, adjuvant therapy compliance, graft patency, and disease recurrence. Follow-up evaluations comprised physical examinations, laboratory testing (including serum tumor markers), and cross-sectional imaging (contrast-enhanced CT/MRI). All follow-up data were collected via telephone interviews conducted by dedicated clinical trial coordinates from our department.

### 2.7 Included indexes

In this study, preoperative variables (white blood cell counts, neutrophil counts, lymphocyte counts albumin, total bilirubin, alanine aminotransferase, carcinoembryonic antigen, carbohydrate antigen 19–9, preoperative neoadjuvant chemotherapy, preoperative biliary drainage), intraoperatie indexes (surgical procedure, operation time, blood loss, blood transfusion), pathological data (pathological diagnosis, tumor differentiation, resection margin, lymph node metasis), patency rate of reconstructed vessels, postoperative complications and overall prognosis were obtained from the electronic medical record system and follow-up results. Postoperative pancreatic fistula was defined as a drain fluid amylase activity greater than three times the upper limit of normal on or after postoperative day 3, and was classified as biochemical leak, Grades B and C according to the ISGPS definition (19). Postpancreatectomy hemorrhage according to ISGPS guidelines (20), comprising extraluminal hemorrhage (bleeding into the peritoneal cavity identified via abdominal drains or unexplained hemodynamic instability) and intraluminal hemorrhage (gastrointestinal bleeding presenting as hematemesis, melena, or hematochezia). Abdominal infection was defined by clinical signs of sepsis or peritonitis combined with positive microbiological culture of abdominal drainage. Portal vein thrombosis was defined as luminal obstruction of the portal vein or AVG conduit on contrast-enhanced CT (21). Pulmonary infection was defined as clinical respiratory symptoms accompanied by new pulmonary infiltrates on chest CT. Reconstructed vessel stenosis was defined as a 50% reduction in luminal cross-sectional area compared with baseline cross-sectional area on POD 7, and was categorized into four grades: no stenosis, slight stenosis, moderate stenosis and severe stenosis. The severity of all other complications was assessed using the Clavien–Dindo classification (22).

### 2.8 Statistical analysis

Continuous variables were assessed for normality; normally distributed data were expressed as means ± standard deviation and compared using independent-samples *t*-tests when comparing two groups of continuous data, whereas one-way variance analysis was applied when comparing multi-group continuous data. Non-normally distributed data were expressed as medians (interquartile range). The rank sum test was selected if measurement data did not obey a normal distribution. Categorical variables were presented as frequencies and percentages were compared using the chi-square test or Fisher’s exact test, as appropriate. If the missing data rate for a continuous variable was less than 20%, missing values were imputed using mean imputation for normally distributed variables or median imputation for non-normally distributed variables. For survival analysis, overall survival and disease-free survival curves were constructed using the Kaplan–Meier method, and differences between survival distributions were compared using the log-rank test. Univariate logistic regression was applied as multivariate analysis to identify independent risk factors. Univariate and multivariate Cox proportional hazards regression models were used to identify independent risk factors associated with survival outcomes. All statistical analyses were performed using SPSS Software (version 23.0; IBM Corporation, Armonk, NY, United States) and R (version 4.5.0; R Foundation for Statistical Computing, Vienna, Austria), with two-sided *p* values < 0.05 considered statistically significant.

## 3 Results

### 3.1 General condition of included patients

A total of 187 patients were enrolled in this study, including 103 males and 84 females. The mean age of the cohort was 61.6 ± 10.9 years. The pathological diagnoses among enrolled patients comprised pancreatic ductal adenocarcinoma (*n* = 126), cholangiocarcinoma (*n* = 30, including 24 cases of distal cholangiocarcinoma, 3 cases of hilar cholangiocarcinoma, and 3 cases of gallbladder carcinoma), pancreatic adenosquamous carcinoma (*n* = 16), autoimmune and chronic pancreatitis (*n* = 7), pancreatic neuroendocrine tumors (*n* = 4), pancreatic solid pseudopapilloma neoplasm (*n* = 1), pancreatic sarcomatoid carcinoma (*n* = 1), and pancreatic serous cystadenomas (*n* = 2). Detailed baseline characteristics are summarized in Table 1.

**Table 1:** Preoperative general condition of included patients.

Item/Index		Result
Age	Years, (Mean ± SD)	61.6 ± 10.9
Gender	(Male/Female), *n* (%)	103 (55.1)/84 (44.9)
Smoking history	(Yes/No), *n* (%)	54(28.9)/133 (71.1)
Hypertension	(Yes/No), *n* (%)	57 (30.5)/130 (69.5)
Coronary heart disease	(Yes/No), *n* (%)	11 (5.9)/176 (94.1)
Cerebral infarction	(Yes/No), *n* (%)	11 (5.9)/176 (94.1)
Diabetes	(Yes/No), *n* (%)	49 (26.2)/138 (73.8)
Family history	(Yes/No), *n* (%)	3 (1.6)/184 (98.4)
Initial symptoms		
Jaundice	*n* (%)	78 (41.7)
Abdominal pain	*n* (%)	73 (39.0)
Physical examination	*n* (%)	19 (10.2)
Atypical digestive symptoms	*n* (%)	15(8.0)
Tumor recurrence	*n* (%)	2 (1.1)
WBC	×10^9^/L, (Mean ± SD)	6.18 ± 2.39
ALB	g/L, (Mean + SD)	38.0 ± 5.35
ALT	U/L, M (P25, P75)	47 (18.5, 125.5)
AST	U/L, M (P25, P75)	36 (20.0, 98.5)
TBil	μm/L, M (P25, P75)	28.2 (10.5, 132.2)
GGT	U/L, M (P25, P75)	155 (27.5, 409.0)
CA19-9	U/ml, M (P25, P75)	243 (27.8, 1159.0)
CEA	ng/ml, M (P25, P75)	2.3 (1.5, 4.6)
Preoperative biliary drainage	(Yes/No), *n* (%)	46 (24.6)/141 (75.4)
Preoperative neoadjuvant chemotherapy	(Yes/No), *n* (%)	12 (6.4)/175 (93.6)
Paclitaxel + Gemcitabine	*n* (%)	5 (2.7)
Paclitaxel + Tegafur	*n* (%)	3 (1.6)
Leucovorin + Fluorouracil + Irinotecan + Oxaliplatin	*n* (%)	3 (1.6)
Paclitaxel + Gemcitabine + Nimotuzumab	*n* (%)	1 (0.5)

SD, Standard deviation; M, Median; WBC, White blood cell; ALB, Albumin; ALT, Alanine transaminase; AST, Aspartic transaminase; TBil, Total bilirubin; GGT, Gamma-glutamyl transpeptidase; CA19-9, Carbohydrate antigen; CEA, Carcino-embryonic antigen.

### 3.2 Intraoperative condition and pathological results

Included patients underwent pancreaticoduodenectomy, total pancreatectomy, or distal pancreatectomy according to their preoperative diagnosis and tumor location. Intraoperative vascular resection and reconstruction with AVGs were performed in all patients commensurate with the extent of vascular involvement and resulting defect. All patients underwent successful surgical resection, and no intraoperative deaths occurred. Among patients with malignant disease, R0 resection was achieved in 166 patients, yielding an overall R0 resection rate of 92.2%. For patients with pancreatic cancer and cholangiocarcinoma, the R0 resection rates were 92.3% (132/143) and 90.0% (27/30), respectively, demonstrating high R0 resection rates in patients undergoing AVG-based reconstruction regardless of tumor pathology. Detailed intraoperative outcomes are summarized in Table 2.

**Table 2:** Surgical, pathological, and postoperative data of included patients.

Item/Index		Result
Surgical treatment		
Pancreaticoduodenectomy	*n* (%)	129 (69.0)
Distal pancreatectomy	*n* (%)	9 (4.8)
Total pancreaticoduodenectomy	*n* (%)	49 (26.2)
Intraoperative blood loss	ml, M (P25, P75)	800 (500, 1,000)
Blood transfusion	Yes/No; ml, M (P25, P75)	118/69; 800 (0, 800)
Operation time	Hours, (Mean ± SD)	10.66 ± 3.166
Pathological classification		
Pancreatic adenocarcinoma	High/Moderate-High/Moderate/Poor-moderate/Poor; *n* (%)	10 (5.3)/6 (3.2)/82 (43.9)/10 (5.3)/18 (9.6)
Cholangiocarcinoma	*n* (%)	30 (16.0)
Pancreatic adenosquamous carcinoma	*n* (%)	16 (8.6)
Autoimmune and chronic pancreatitis	*n* (%)	7 (3.7)
Pancreatic neuroendocrine neoplasm	*n* (%)	4 (2.1)
Pancreatic serous cystadenoma	*n* (%)	2 (1.1)
Pancreatic solid pseudopapillary neoplasm	*n* (%)	1 (0.5)
Pancreatic sarcomatoid carcinoma	*n* (%)	1 (0.5)
Lymph nodes metastasis		
	Yes, *n* (%)	135 (72.2)
	No, *n* (%)	45 (24.1)
	Benign lesion, *n* (%)	7 (3.7)
Perineural invasion		
	Yes, *n* (%)	176 (94.1)
	No, *n* (%)	4 (2.1)
	Benign lesion, *n* (%)	7 (3.7)
Resection margin		
	R0, *n* (%)	166 (88.8)
	R1, *n* (%)	14 (7.5)
	Benign lesion, *n* (%)	7 (3.7)
Postoperative adjuvant chemotherapy		
	No, *n* (%)	108 (57.8)
	Paclitaxel + Gemcitabine, *n* (%)	20 (10.7)
	Tegafur, *n* (%)	19 (10.2)
	Gemcitabine + Tegafur, *n* (%)	13 (7.0)
	Gemcitabinie, *n* (%)	8 (4.3)
	Capecitabine, *n* (%)	7 (3.7)
	Gemcitabine + Capecitabine, *n* (%)	3 (1.6)
	Paclitaxel + Tegafur, *n* (%)	3 (1.6)
	Cis-platinum + Gemcitabine, *n* (%)	2 (1.1)
	Oxaliplatin + Gemcitabine, *n* (%)	2 (1.1)
	Oxaliplatin + Capecitabine, *n* (%)	2 (1.1)

SD, Standard deviation; M, Median.

### 3.3 Protocol and technique in vascular resection and reconstruction

According to the Chaoyang classification, the extent of vascular invasion was categorized intraoperatively into Type II (*n* = 77), Type III (*n* = 95), and Type IV (*n* = 15) based on intraoperative evaluation. The anatomical distribution of vascular invasion is detailed in Table 3.

**Table 3:** Location of vascular resection and reconstruction.

Location of vascular resection and reconstruction		Application of allograft vessels
PV, *n* (%)	25 (13.9)	Tubular allograft vessels with no branches
SMV, *n* (%)	50 (26.7)	Allograft vessels with or without branches
PV-SMV-SV, *n* (%)	95 (50.8)	Branched allograft vessels or spliced allograft vessels
PV-SMV branches-SV, *n* (%)	15 (8.0)	
Left RV and IVC, *n* (%)	2 (1.1)	Branched allograft vessels, spliced allograft vessels

PV, Portal vein; SMV, Superior mesenteric vein; SV, Splenic vein; RV, Renal vein; IVC, Inferior vena cava.

**Figure 3:**
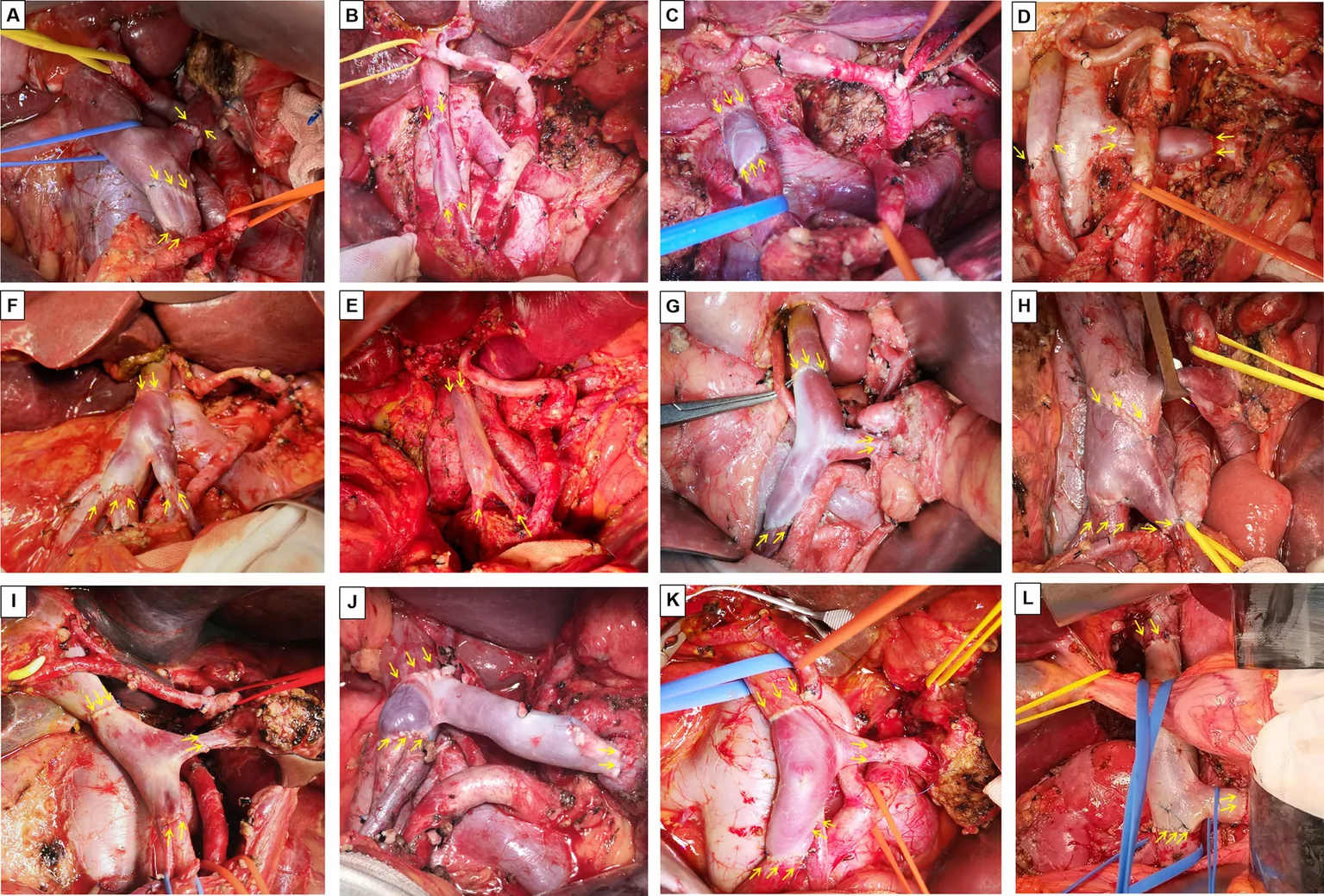
Intra-operative figure of application of tubular or branched AVGs in reconstructing invaded abdominal veins. **(A–D)** Application of tubular AVGs in reconstructing SMV **(A)**, SMV–PV **(B)**, PV **(C)**, and LRV **(D)**. **(E,F)** Application of single or double Y-shape branched AVGs in reconstructing SMV-PV with its branches **(E)**, SMV–PV with its branches and colic vein **(F)**. **(G–I)** Application of T-shape AVGs in reconstructing SMV–SV–PV **(G,I)** and SMV–PV with its branches **(H)**. **(J–L)** Application of L-shape AVGs in reconstructing SMV–SV–PV **(J)**, SMV–SV–PV with SMV branches **(K)**, and IVC–LRV **(L)**. AVG, Allogeneic venous grafts; PV, Portal vein; SMV, Superior mesenteric vein; LRV, Left renal vein; IVC, Inferior vena cava; Yellow arrow refers to anastomosis of vascular reconstruction.

**Figure 4:**
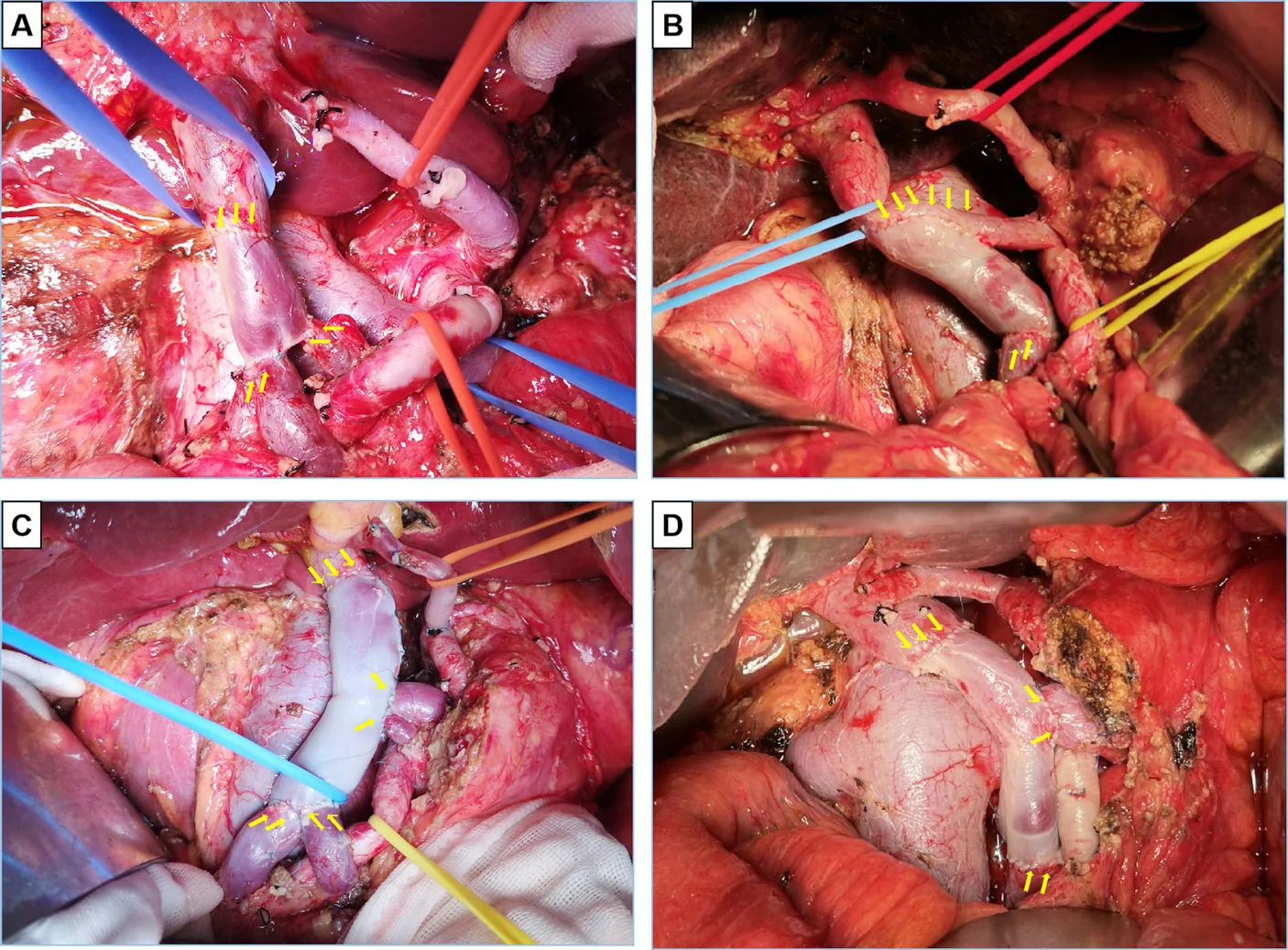
Intraoperative figure of application of trimmed I-shape tubular AVG in reconstructing the portal venous system. **(A)** Reconstructing SMV and its branches with the trimmed distal end of I-shape tubular AVG; **(B)** Reconstructing the confluence of SMV and SV with the trimmed proximal end of I-shape tubular AVG; **(C)** Reconstructing SMV and its branches with the trimmed distal end while reconstructing SV and IMV with the trimmed side wall of I-shape tubular AVG; **(D)** Reconstructing SV with the trimmed side wall of I-shape tubular AVG. AVG, Allogeneic venous graft; SMV, Superior mesenteric vein; SV, Splenic vein; IMV, Inferior mesenteric vein; Yellow arrow refers to anastomosis of vascular reconstruction.

**Figure 5:**
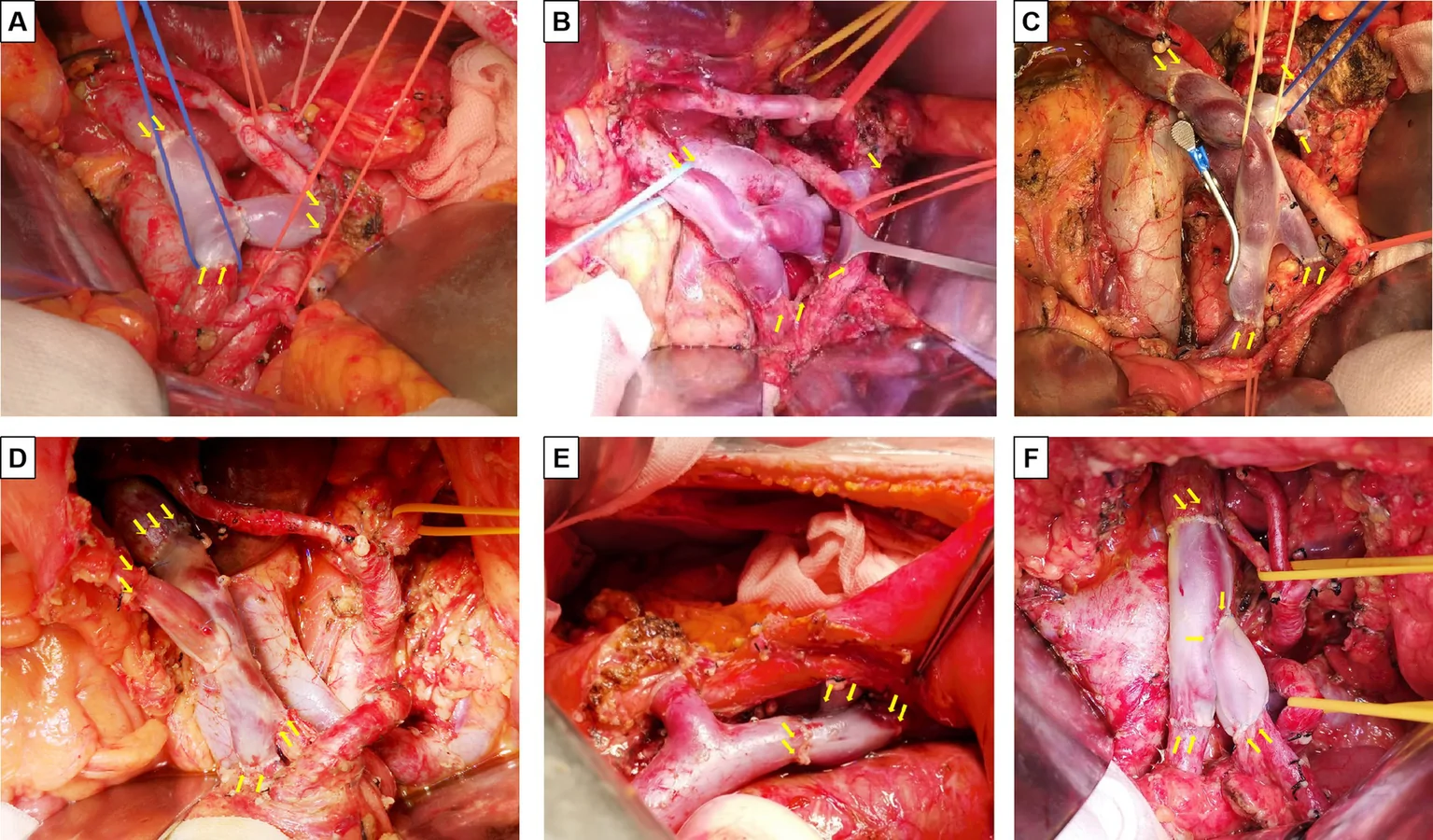
Intra-operative figure of application of spliced AVG in reconstructing abdominal veins. **(A)** Reconstructing SMV–SV–PV with spliced AVG constitute of two I-shape tubular vessels; **(B)** reconstructing SMV–SV–PV and the branch of SMV with spliced AVGs constitute of I-shape and T-shape AVGs; **(C)** reconstructing SMV–SV–PV–IMV and the branch of SMV with spliced AVG constitute of Y-shape and T-shape AVGs; **(D)** reconstructing PV-SMV, the branch of SMV and MCV with spliced AVG constitute of I-shape and Y-shape AVGs; **(E)** reconstructing SMV and JV with spliced AVG constitute of I-shape and L-shape AVGs; **(F)** reconstructing PV-SMV and two branches of SMV with spliced AVG constitute of I-shape and short L-shape AVGs. AVG, Allogeneic venous graft; SMV, Superior mesenteric vein; SV, Splenic vein; PV, Portal vein; IMV, Inferior mesenteric vein; MCV, Middle colic vein; JV, Jejunum vein; Yellow arrow refers to anastomosis of vascular reconstruction.

In the study cohort, primary sites of AVG reconstruction included the portal vein–superior mesenteric PV-SMV-SV confluence (*n* = 110) including 15 cases requiring additional SMV branch reconstruction, the isolated SMV (*n* = 50), the main PV trunk (*n* = 25), the IVC combined with the RV (*n* = 1), and the LRV (*n* = 1), as illustrated in Figures 3–5. Regarding the technical configurations of vascular reconstruction, 75 patients underwent reconstructed using straight tubular AVGs (I-configuration), including trimmed I-shape AVGs, 112 patients applied branched AVGs for vascular reconstruction including 74 cases of single-branch AVGs (47 cases of Y-shape, 15 cases of T-shape and 12 cases of L-shape) and 38 cases of spliced AVGs with multi-shape AVGs (16 cases of spliced T-shape and L-shape AVGs, 10 cases of spliced double I-shape AVGs, 6 cases of spliced T-shape and I-shape AVGs, 4 cases of spliced Y-shape and I-shape AVGs, and 2 cases of Y-shape and T-shape AVGs).

### 3.4 Postoperative complications after AVGs reconstruction

Postoperative complications occurred in 62 patients, yielding an overall complication rate of 33.2%, and the complications included postoperative pancreatic fistula (*n* = 24), abdominal infection (*n* = 24), abdominal hemorrhage (*n* = 11), portal vein thrombosis (*n* = 2), and pulmonary infection (*n* = 1). All 11 cases of postoperative abdominal hemorrhage occurred secondary to Postoperative pancreatic fistula or bile leakage, which caused erosion of adjacent vessels and subsequent bleeding. There was no bleeding originating from anastomosis of AVGs reconstruction. 13 of 187 (7.0%) patients died within the perioperative period, including 7 cases of abdominal hemorrhage, 5 cases of abdominal infection, and 1 case of pulmonary infection. After categorizing the patients according to the type of AVGs for vascular reconstruction, the complications, severe complications (Clavien-Dindo grade ≥3), reoperation, and in-hospital stay were not statistically significant (Table 4).

**Table 4:** Comparison of postoperative complications among different types of AVGs.

Variables	Tubular AVGs (*n* = 75)	Single-branch AVGs (*n* = 74)	spliced AVGs (*n* = 38)	*p* Value
Complications, *n* (%)	26 (34.7)	24 (32.4)	12 (31.6)	0.934
Severe complications*, *n* (%)	18 (24.0)	13 (17.6)	10 (26.3)	0.487
Perioperative death, *n* (%)	7 (9.3)	4 (5.4)	2 (5.3)	0.701
Grade B pancreatic fistula, *n* (%)	7 (9.3)	4 (5.4)	3 (7.9)	0.674
Grade C pancreatic fistula, *n* (%)	5 (6.7)	4 (5.4)	1 (2.6)	0.775
Abdominal hemorrhages, *n* (%)	6 (8.0)	3 (4.1)	2 (5.3)	0.672
Abdominal infection, *n* (%)	11 (14.7)	8 (10.8)	5 (13.2)	0.779
Pulmonary infection, *n* (%)	1 (1.3)	0 (0.0)	0 (0.0)	1.000
Portal vein thrombosis, *n* (%)	1 (1.3)	1 (1.4)	0 (0.0)	1.000
Re-operation, *n* (%)	10 (13.3)	6 (8.1)	5 (13.2)	0.549
In-hospital stay (days)	23.0 (18.0, 35.0)	22.5 (17.0, 34.0)	23 (17.0, 39.0)	0.867

AVG, Allogeneic vascular graft; *Severe complication was defined as Clavien-Dindo grade ≥3.

### 3.5 Incidence of reconstructed vessel stenosis after AVGs reconstruction

**Figure 6:**
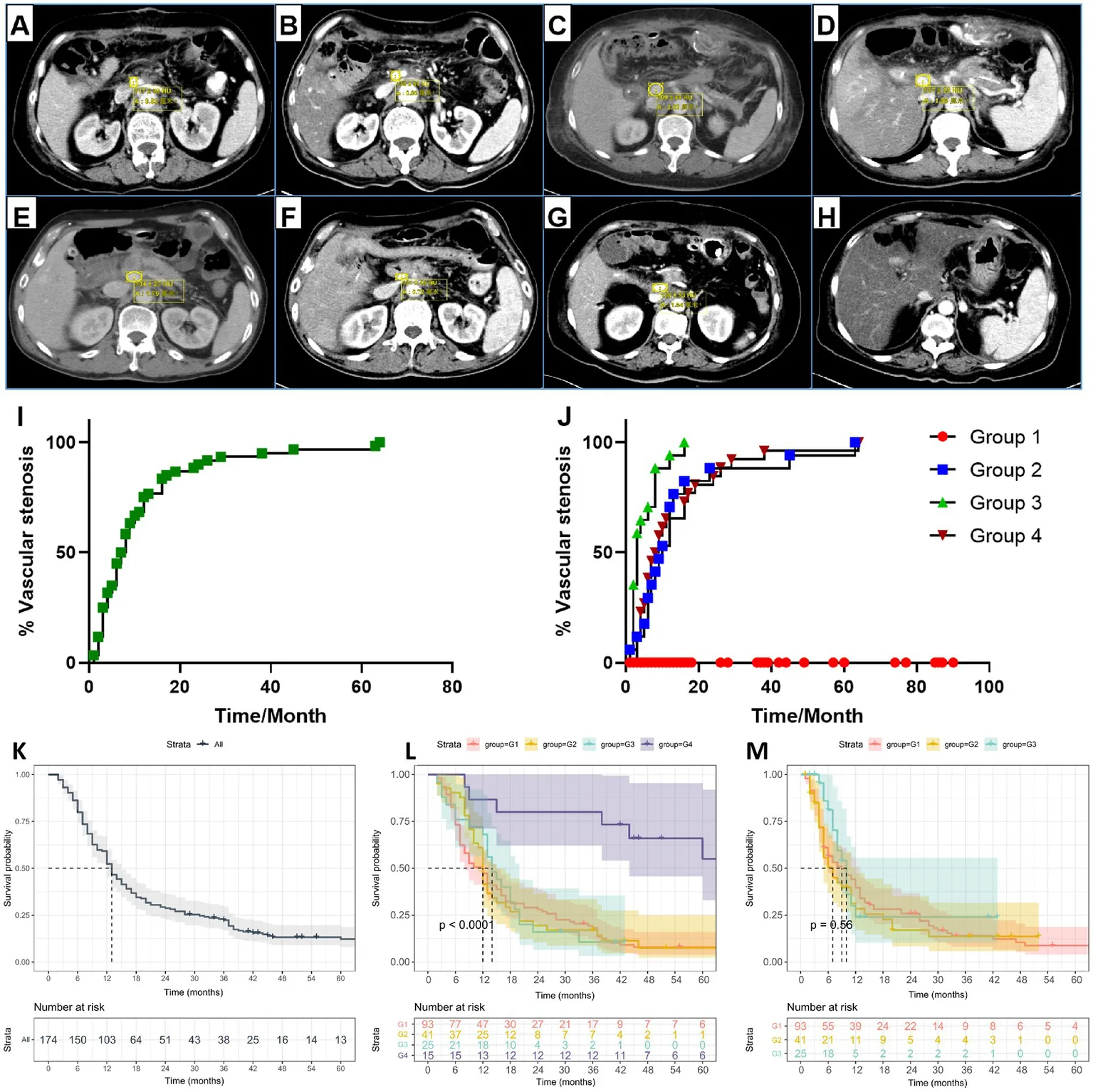
Follow-up and survival condition of patients receiving vascular reconstruction with AVG. **(A,B)** Abdominal enhanced CT of a patient undergoing SMV reconstruction with AVG at postoperative 1 month **(A)** and 6 months **(B)** indicating no reconstructed vessel stenosis (AVG lumen area were 0.83 and 0.86 cm^2^ respectively); **(C,D)** Abdominal enhanced CT of a patient undergoing PV reconstruction with AVG at postoperative 1 week **(C)** and 21 months **(D)** indicating slight reconstructed vessel stenosis (AVG lumen area were 2.2 and 1.65 cm^2^ respectively); **(E,F)** Abdominal enhanced CT of a patient undergoing PV reconstruction with AVG at postoperative 1 week **(E)** and 3 months **(F)** indicating median reconstructed vessel stenosis (AVG lumen area were 1.79 and 0.72 cm^2^ respectively); **(G,H)** Abdominal enhanced CT of a patient undergoing PV reconstruction with AVG at postoperative 1 week **(G)** and 11 months **(H)** indicating severe reconstructed vessel stenosis (AVG lumen area at postoperative 1 week was 1.54 cm^2^ while the main portal vein developed CTPV and cannot be observed at postoperative 11 months); **(I,J)** Incidence curve of overall vascular stenosis after AVGs reconstruction **(I)** and different degree of vascular stenosis after AVGs reconstruction **(J)**, group 1, group 2, group 3, group 4 refer to no, slight, moderate, severe reconstructed vessel stenosis; **(K,L)** The survival curve of overall survival in all included patients **(K)** and patients with different tumor types **(L)** after excluding perioperative death. **(M)** The survival curve of disease-free survival in all included patients with malignant tumors. Group 1, group 2, group 3, group 4 refer to malignant tumor at pancreatic head and uncinate process, malignant tumor at pancreatic neck, body and tail, cholangiocarcinoma including gallbladder carcinoma, other lesion, respectively. AVG, Allogeneic venous graft; CT, Computerized tomography.

According to the cross-sectional luminal area measured on abdominal-enhanced CT, graft vascular stenosis was categorized into no stenosis (Figures 6A,B), mild stenosis (Figures 6C,D), moderate stenosis (Figures 6E,F), and severe stenosis (Figures 6G,H). A total of 46 of 174 evaluable patients developed moderate-to-severe graft stenosis following vascular resection and AVGs reconstruction, representing an overall cumulative incidence rate of 26.4%. The cumulative incidence curve of reconstructed vascular stenosis (Figures 6I,J) showed stenosis rates of 18.4%, 21.8%, and 33.3% at 1-year, 2-year, and 3-year after surgery, respectively. For pancreatic cancer patients, 38 (28.1%) patients developed moderate-to-severe stenosis, with a 1-year, 2-year, and 3-year stenosis rate of 27.2%, 40.7%, and 43.8%, respectively. For cholangiocarcinoma, moderate–severe stenosis was observed in 6 (24.0%) patients. All cholangiocarcinoma patients developed stenosis within 1 year postoperatively, with a 1-year stenosis rate of 48.1%. The univariate analysis of potential risk factors for moderate to severe reconstructed vessel stenosis included diabetes history, preoperative albumin, preoperative CA19-9, and surgical procedure. Further multivariate analysis indicated that preoperative albumin (OR: 1.097, 95%*CI*: 1.019–1.180, *p* = 0.013), preoperative CA19-9 (OR: 1.258, 95%*CI*: 1.074–1.474, *p* = 0.004) and surgical procedure (OR: 3.512, 95%*CI*: 1.387–8.894, *p* = 0.008) were independent risk factor for postoperative moderate to severe reconstructed vessels stenosis (Supplementary Table 1). Since vascular stenosis may cause severe complications that impact postoperative prognosis, we further evaluated the correlation between vascular stenosis. Regarding overall prognosis, the OS time of patients with and without postoperative vascular stenosis was 12 and 13 months in the entire cohort, with 1-year, 2-year, and 3-year OS rates of 43.5%, 19.6%, 8.7%, and 55.5%, 32.0%, 14.0% (*p* = 0.129). For pancreatic cancer patients, the OS time of patients with and without postoperative vascular stenosis was 10 and 13 months, with 1-year, 2-year, and 3-year OS rates of 36.8%, 18.4%, 2.6%, and 56.7%, 29.9%, 10.4% (*p* = 0.052). As for DFS, the DFS time of patients with and without postoperative vascular stenosis was 8 and 10 months in the entire cohort after excluding benign lesions, with 1-year, 2-year, and 3-year OS rates of 28.4%, 14.2%, 7.1%, and 39.2%, 28.1%, 13.7% (*p* = 0.064). The DFS time of patients with and without postoperative vascular stenosis was 5 and 10 months in pancreatic cancer patients, with 1-year, 2-year, and 3-year OS rates of 30.0%, 15.0%, 7.5%, and 39.4%, 26.7%, 16.6% (*p* = 0.062).

### 3.6 Long-term prognosis after AVGs reconstruction

All included patients had complete follow-up data, and the median follow-up time was 88.0 months. During the postoperative follow-up period, 13 patients died within the perioperative period and were excluded from further survival analysis. A total of 79 (42.2%) patients received adjuvant chemotherapy, and the regimens are shown in Table 2. The survival analysis in the remaining 174 patients indicated a median overall survival time of 13 months and a 5-year overall survival rate of 12.2% (Figure 6K). The survival of OS for patients with pancreatic adenocarcinoma (pancreatic head, uncinate process, neck, body, and tail), cholangiocarcinoma, and other lesions is shown in Figure 6L. For pancreatic cancer patients, the median OS was 13 months, and the 1-year and 5-year survival rates were 51.1% and 8.2%, respectively. For cholangiocarcinoma patients, the median OS was 12 months, and the 1-year and 5-year survival rates were 40.0% and 8.0%, respectively. According to univariate and multivariate analysis, age (HR: 1.031, 95%*CI*: 1.016–1.046, *p* < 0.001), preoperative CA19-9 (HR: 1.114, 95%*CI*: 1.036–1.197, *p* = 0.004), operation time (HR: 1.059, 95%*CI*: 1.005–1.115, *p* = 0.033), lymph node metastasis (HR: 1.679, 95%*CI*: 1.135–2.483, *p* = 0.010), postoperative complication (HR: 2.190, 95%*CI*: 1.519–3.157, *p* < 0.001) and postoperative chemotherapy (HR: 0.708, 95%*CI*: 0.509–0.985, *p* = 0.040) were independently correlated with postoperative overall survival in patients with malignant cancers (Supplementary Table 2).

Among all patients with malignant disease, a total of 111 patients experienced tumor recurrence during follow-up, representing an overall recurrence rate of 64.2%. After excluding patients who experienced perioperative mortality and had benign lesions, the median disease-free survival (DFS) duration for the remaining 159 cases was 9 months, with 1-year, 2-year, 3-year, and 5-year DFS rates of 36.8%, 25.0%, 16.6%, and 12.7%, respectively. The liver and local residual pancreatic bed were the most common sites of postoperative recurrence, accounting for 82 and 29 patients among all recurrence patients. The survival curves of DFS in patients with pancreatic adenocarcinoma (pancreatic head, uncinate process, neck, body, and tail), cholangiocarcinoma, and other malignant cancers are shown in Figure 6M. The median DFS time of patients with pancreatic cancer and cholangiocarcinoma was 8 and 10 months, respectively. The 1-year and 5-year DFS rates of pancreatic cancer and cholangiocarcinoma were 36.7%, 9.5%, and 30.1%, 24.0%, respectively. As shown in Supplementary Table 3, only preoperative CEA (HR: 1.023, 95%*CI*: 1.004–1.042, *p* = 0.017) and postoperative complication (HR: 1.562, 95%*CI*: 1.060–2.302, *p* = 0.024) were independent risk factors for postoperative disease-free survival in patients with malignant cancers.

## 4 Discussion

Vascular invasion is frequently encountered in advanced visceral malignancies (23). With advancements in vascular, microsurgery, and transplant surgical techniques, vascular resection and reconstruction techniques have enabled an increasing number of such patients to undergo radical surgery and increased the success rate of surgery (24, 25). However, the selection of vascular reconstruction material and technique remains a matter of debate. Current research mainly discusses vascular reconstruction techniques to restore continuity across different types of vascular invasion and defect, aiming to provide an opportunity for radical surgery for patients in need of vascular resection and reconstruction due to vascular invasion.

The application of AVGs was first proposed by Watts in 1907 (26). Over the following century, AVGs have been widely applied in the surgical management of several abdominal diseases. Over the past decade, our center has been exploring the application of AVGs in radical resection of borderline resectable pancreatic cancer (BRPC), proposing a PV system reconstruction-oriented assessment of resectability and demonstrating that radical resection can be achieved through accurate preoperative evaluation and sufficient surgical preparation if the PV system can be safely reconstructed. In addition, we also put forward the Chaoyang vascular invasion classification and established a systematic AVGs reconstruction protocol for radical surgery of abdominal tumors with vascular invasion. Various indications and methods of AVGs application in vascular reconstruction were designed to meet the diverse scenarios of vascular defect in different surgical conditions. Currently, it has been widely adopted in the radical surgery of various abdominal tumors, including pancreatic cancer, cholangiocarcinoma, ampullary tumor, retroperitoneal tumor, renal cancer, stromal tumor, and smooth muscle tumor, achieving satisfying clinical outcomes.

In current research, we explored vascular reconstruction methods for pancreatic cancer with different types and extents of vascular invasion. In clinical practice, the extent of vascular invasion observed intraoperatively frequently exceeds preoperative imaging estimates, urging the necessity to prepare AVGs for BRPC patients with suspected vascular invasion in advance. Currently, extended radical pancreaticoduodenectomy with PV system resection has been increasingly accepted in specialized pancreatic surgery centers (27, 28). However, there is still no clear consensus on how to reconstruct the vascular defects and which grafts to choose. In terms of the source of AVGs, researchers obtained and preserved iliac vessels and splenic artery of donors relying on the organ transplant center and found that vascular reconstruction with allograft veins had a high long-term patency rate, good histocompatibility, and the necessity of lifelong anticoagulants after surgery compared with allograft arteries and biological vascular materials (29, 30). The postoperative complications and long-term patency rates of reconstructed vessels after PV system reconstruction in BRPC are consistent with previous reports in the literature (9, 31). However, since artificial vessels or autologous vein grafts were not routinely applied in our center and the number of relevant patients was rather limited, we were not able to compare the outcome after AVGs reconstruction with other vascular reconstruction material directly. Therefore, these findings should be interpreted cautiously.

We designed different types of AVGs application in vascular reconstruction according to the extent of vascular invasion and tumor location. Direct end-to-end anastomosis can be adopted when only 0.5–1 cm of vessel is resected. It can also be considered for large vascular defects if tension-free anastomosis can be achieved after full mobilization of both proximal and distal ends. However, according to our clinical practice, we recommend tubular (I-shape) AVGs instead of direct end-to-end anastomosis under such conditions in patients with malignant tumors since it allows a larger extent of vascular resection to facilitate more complete clearance of vascular margin, adjacent connective tissue, and local lymph nodes, thereby reducing the risk of recurrence. Therefore, we advocate the use of tubular (I-shape) AVGs for 1–4 cm tubular vascular defect proximal or distal to the confluence of PV system. However, when the extent of vascular defect exceeds 3 cm or involves the confluence of the PV system, it is difficult to perform direct end-to-end anastomosis after PV and (or) SMV resection. Under this scenario, AVGs interposition can reduce the anastomotic tension and may potentially lower the risk of postoperative anastomotic stenosis and leakage (32, 33).

However, the actual extent of tumor invasion and the individual anatomical variation often lead to resection of PV system confluence or major venous branches, causing challenges for vascular reconstruction. Under this circumstance, tubular AVGs cannot fulfill the requirements of vascular reconstruction since reconstructing the main trunk of the PV with an I-shape AVG only is associated with a higher risk of severe postoperative complications (34, 35). After years of practice, we have summarized a variety of methods and techniques of vascular reconstruction using branched (Y-shape, T-shape, and L-shape) AVGs. In our cohort, the application of branched AVGs achieved a favorable R0 resection rate with acceptable perioperative complication and stenosis rates. Vascular occlusion occurred in only 2 cases of portal vein thrombosis during the perioperative period. Compared with biological vascular materials and autologous vessels, the application of AVGs may help avoid lifelong anticoagulant medication, excessive surgical damage, and insufficient vascular length. However, these potential benefits need further verification due to the uncontrolled study design and lack of direct comparator groups.

According to our experience, Y-shape and I-shape AVGs have the widest range of clinical application and can accommodate various extents of vascular defect. When complete branched AVGs are not available, trimming and combining two or more L-shape or I-shape AVGs into a spliced branched AVG is an effective alternative solution that can restore hemodynamic and vascular compliance comparable to complete Y-shape AVGs. When resecting vessels on both cephalic and podalic sides of PV system confluence or vessels on either side of PV system confluence and SV, these techniques enable complete PV system reconstruction and reduce the risk of regional portal hypertension after surgery while avoiding inadequate resection margins. When important branches such as renal vein, middle colic vein are involved, or when regional blood flow is insufficient caused by the division of the second branch of jejunum artery in patients with low tumor location or stromal tumor, T-shape AVGs can be used to repair the main vessel wall at confluence of main trunk and branches, and the reserved unilateral cecum can reduce local hemodynamic pressure and improve regional blood circulation.

No matter what vascular reconstruction method is chosen, completely reconstructing the PV system, restoring local hemodynamics, and maintaining sufficient organ perfusion are the basic principles of vascular reconstruction (13, 36), and achieving R0 resection is the ultimate goal of such surgery. In some cases, AVGs can also be combined to achieve better PV system reconstruction. Taking the combined application of I-shape and Y-shape AVGs in clinical practice as an example, the combined application of AVGs enables simultaneous reconstruction of SV, inferior mesenteric vein, and middle colic vein at the same time. Therefore, flexible application of AVGs in vascular reconstruction to achieve local hemodynamic stability according to the extent of vascular defect, the vessels to be reconstructed, and the shape of AVGs is the key to improving operative success and long-term prognosis. We believe that the proposed methods and techniques of vascular reconstruction with different types of AVGs may have the potential to increase resectability and improve survival in pancreatic surgery in the future and can be further applied in surgeries of vascular diseases, urological diseases, retroperitoneal tumors, and other diseases.

However, there are several limitations of this study that should be acknowledged. First, as a retrospective, single-center study with a relatively small sample size but including patients over 11 years, selection and chronological biases were inherent, and the generalizability of the proposed methods of vascular reconstruction may be limited and require further verification in other institutions or patient populations. Second, the limited number of patients receiving neoadjuvant chemotherapy was another limitation of our research, which may lead to shorter OS and DFS than anticipated under current standard neoadjuvant regimens and should be addressed in future studies. Third, the included tumor entities were heterogeneous, which may have different biological behaviors and prognosis; however, the limited number of cases in each subgroup precluded meaningful subgroup or comparative analysis. Fourth, although we reported perioperative and long-term outcomes after vascular reconstruction with AVGs, the absence of a control group receiving alternative reconstructive materials limits direct comparison of clinical utility and safety between different vascular reconstruction materials. Thus, the superiority of AVGs cannot be clearly demonstrated from the current data. Fifth, immunological screening for AVG recipients was not performed in our center, so the actual incidence of graft rejection remains unknown. Finally, since the data on complications secondary to AVG stenosis were unavailable currently, we failed to illustrate the impact of AVG stenosis on it. Further research is necessary to fully demonstrate this association. Despite these limitations, our study represents one of the largest single-center experiences with AVGs in pancreatic surgery to date and provides valuable real-world data for clinical practice. Our center is also carrying out prospective clinical research to further confirm its clinical value and safety. With the continuous development of this technique, we believe that indications and methods of vascular reconstruction with AVGs will gradually optimize and continuously improve the long-term prognosis of patients with tumorous vascular invasion.

## 5 Conclusion

Flexible application and combination of different shape AVGs can be utilized to manage various extents of venous defects in pancreatic surgeries. The application of AVGs achieved favorable R0 resection rates and acceptable long-term patency and survival outcomes in our cohort. Still, the superiority of AVGs over alternative reconstructive approaches remained to be verified in further research.

## Data Availability

The raw data supporting the conclusions of this article will be made available by the authors, without undue reservation.
