# Supplementary material for: Utilizing allogeneic venous grafts to solve the problem of vascular defects and reconstruction in pancreatic surgery: a retrospective cohort study
Source: Front Med (Lausanne). 2026 Aug 26;13:1875288. doi: 10.3389/fmed.2026.1875288 (PMC13555790; doi:10.3389/fmed.2026.1875288)
Supplement: Supplementary file 1 [file Data_Sheet_1.DOCX]

**Supplementary material**

**Supplementary Tables**

**Supplementary Table 1. Univariate and multivariate analysis for reconstructed vessel stenosis**

| **Variables** | **Univariate analysis** | | | **Multivariate analysis** | | |
| --- | --- | --- | --- | --- | --- | --- |
|  | **OR** | **95%CI** | **P value** | **OR** | **95%CI** | **P value** |
| Age (years) | 0.974 | 0.946 - 1.003 | 0.082 |  |  |  |
| Sex (Male/Female) | 1.545 | 0.779 - 3.061 | 0.213 |  |  |  |
| Diabetes (Yes/No) | 0.396 | 0.164 - 0.954 | 0.039* | 0.550 | 0.218 - 1.388 | 0.205 |
| Smoking (Yes/No) | 1.347 | 0.663 - 2.735 | 0.410 |  |  |  |
| Preoperative WBC (×10^9^) | 1.080 | 0.942 - 1.239 | 0.269 |  |  |  |
| Preoperative LYM (×10^9^) | 1.098 | 0.671 - 1.797 | 0.710 |  |  |  |
| Preoperative PLT (×10^9^) | 1.000 | 0.997 - 1.004 | 0.827 |  |  |  |
| Preoperative ALB (g/L) | 1.070 | 1.002 - 1.142 | 0.045* | 1.097 | 1.019 - 1.180 | 0.013* |
| Preoperative TB (μmol/L) | 1.002 | 0.999 - 1.006 | 0.257 |  |  |  |
| Preoperative CEA (ng/ml) | 1.010 | 0.968 - 1.053 | 0.650 |  |  |  |
| Preoperative CA19-9 (U/L) | 1.189 | 1.028 - 1.376 | 0.019* | 1.258 | 1.074 - 1.474 | 0.004* |
| Preoperative biliary drainage (Yes/No) | 0.469 | 0.194 - 1.138 | 0.094 |  |  |  |
| Diagnosis (Pancreatic cancer/Others) | 1.629 | 0.695 - 3.815 | 0.261 |  |  |  |
| Surgical Procedure (PD/Others) | 2.610 | 1.130 - 6.026 | 0.025* | 3.512 | 1.387 - 8.894 | 0.008* |
| Operation time (hours) | 0.986 | 0.887 - 1.097 | 0.799 |  |  |  |
| Intraoperative blood loss (per 100ml) | 0.989 | 0.944 - 1.035 | 0.627 |  |  |  |
| Blood transfusion (Yes/No) | 1.067 | 0.535 - 2.129 | 0.854 |  |  |  |
| Resection margin (R1/Others) | 4.570 | 0.581 - 35.935 | 0.149 |  |  |  |
| Lymph node metastasis (Yes/No) | 1.360 | 0.661 - 2.799 | 0.404 |  |  |  |
| Nerve invasion (Yes/No) | 1.500 | 0.312 - 7.208 | 0.613 |  |  |  |
| Chao-yang Classification (III-IV/II) | 1.007 | 0.512 - 1.980 | 0.984 |  |  |  |
| Reconstruction procedure  (Branched AVGs/Tubular AVGs) | 1.192 | 0.600 - 2.368 | 0.616 |  |  |  |
| Postoperative complication (Yes/No) | 1.180 | 0.575 - 2.419 | 0.652 |  |  |  |
| Postoperative chemotherapy (Yes/No) | 1.202 | 0.615 - 2.349 | 0.590 |  |  |  |

(WBC: White blood cell; LYM: Lymphocyte; PLT: Platelet; ALB: Albumin; TB: Total bilirubin; CEA: Carcinoembryonic antigen; CA19-9: Carbohydrate antigen; PD: Pancreaticoduodenectomy; AVGs: Allogeneic vascular grafts; * *P* < 0.05)

**Supplementary Table 2. Univariate and multivariate analysis for overall survival in malignant tumor**

| **Variables** | **Univariate analysis** | | | **Multivariate analysis** | | |
| --- | --- | --- | --- | --- | --- | --- |
|  | **HR** | **95%CI** | **P value** | **HR** | **95%CI** | **P value** |
| Age (years) | 1.017 | 1.003 - 1.031 | 0.020* | 1.031 | 1.016 - 1.046 | < 0.001* |
| Sex (Male/Female) | 1.232 | 0.904 - 1.679 | 0.187 |  |  |  |
| Diabetes (Yes/No) | 1.053 | 0.750 - 1.478 | 0.767 |  |  |  |
| Smoking (Yes/No) | 1.295 | 0.928 - 1.808 | 0.129 |  |  |  |
| Preoperative WBC (×10^9^) | 1.024 | 0.960 - 1.092 | 0.477 |  |  |  |
| Preoperative LYM (×10^9^) | 0.878 | 0.696 - 1.106 | 0.269 |  |  |  |
| Preoperative PLT (×10^9^) | 1.001 | 0.999 - 1.003 | 0.240 |  |  |  |
| Preoperative ALB (g/L) | 0.987 | 0.957 - 1.019 | 0.436 |  |  |  |
| Preoperative TB (μmol/L) | 1.000 | 0.999 - 1.002 | 0.574 |  |  |  |
| Preoperative CEA (ng/ml) | 1.013 | 0.995 - 1.031 | 0.162 |  |  |  |
| Preoperative CA19-9 (U/L) | 1.118 | 1.045 - 1.196 | 0.001* | 1.114 | 1.036 - 1.197 | 0.004* |
| Preoperative biliary drainage (Yes/No) | 1.271 | 0.891 - 1.813 | 0.185 |  |  |  |
| Diagnosis (Pancreatic cancer/Others) | 1.181 | 0.794 - 1.757 | 0.411 |  |  |  |
| Surgical Procedure (PD/Others) | 0.808 | 0.581 - 1.124 | 0.205 |  |  |  |
| Operation time (hours) | 1.052 | 1.001 - 1,106 | 0.045* | 1.059 | 1.005 - 1.115 | 0.033* |
| Intraoperative blood loss (per 100ml) | 1.015 | 0.995 - 1.037 | 0.144 |  |  |  |
| Blood transfusion (Yes/No) | 1.357 | 0.986 - 1.866 | 0.061 |  |  |  |
| Resection margin (R1/Others) | 1.123 | 0.637 - 1.982 | 0.688 |  |  |  |
| Lymph node metastasis (Yes/No) | 1.531 | 1.049 - 2.234 | 0.027* | 1.679 | 1.135 - 2.483 | 0.010* |
| Nerve invasion (Yes/No) | 2.487 | 0.347 - 17.844 | 0.365 |  |  |  |
| Chao-yang Classification (III-IV/II) | 1.054 | 0.773 - 1.439 | 0.739 |  |  |  |
| Reconstruction procedure  (Branched AVGs/Tubular AVGs) | 0.867 | 0.635 - 1.185 | 0.371 |  |  |  |
| Postoperative complication (Yes/No) | 1.861 | 1.343 - 2.578 | < 0.001* | 2.190 | 1.519 - 3.157 | < 0.001* |
| Postoperative chemotherapy (Yes/No) | 0.582 | 0.426 - 0.795 | 0.001* | 0.708 | 0.509 - 0.985 | 0.040* |
| Postoperative vascular stenosis (Yes/No) | 1.172 | 0.820 - 1.676 | 0.384 |  |  |  |

(WBC: White blood cell; LYM: Lymphocyte; PLT: Platelet; ALB: Albumin; TB: Total bilirubin; CEA: Carcinoembryonic antigen; CA19-9: Carbohydrate antigen; PD: Pancreaticoduodenectomy; AVGs: Allogeneic vascular grafts; * *P* < 0.05)

**Supplementary Table 3. Univariate and multivariate analysis for disease-free survival in malignant tumor**

| **Variables** | **Univariate analysis** | | | **Multivariate analysis** | | |
| --- | --- | --- | --- | --- | --- | --- |
|  | **HR** | **95%CI** | **P value** | **HR** | **95%CI** | **P value** |
| Age (years) | 1.008 | 0.992 - 1.023 | 0.341 |  |  |  |
| Sex (Male/Female) | 1.238 | 0.872 - 1.758 | 0.233 |  |  |  |
| Diabetes (Yes/No) | 1.114 | 0.754 - 1.645 | 0.588 |  |  |  |
| Smoking (Yes/No) | 1.428 | 0.979 - 2.083 | 0.064 |  |  |  |
| Preoperative WBC (×10^9^) | 1.045 | 0.975 - 1.120 | 0.216 |  |  |  |
| Preoperative LYM (×10^9^) | 1.001 | 0.774 - 1.295 | 0.994 |  |  |  |
| Preoperative PLT (×10^9^) | 1.001 | 0.999 - 1.003 | 0.491 |  |  |  |
| Preoperative ALB (g/L) | 1.020 | 0.983 - 1.057 | 0.299 |  |  |  |
| Preoperative TB (μmol/L) | 1.000 | 0.998 - 1.002 | 0.713 |  |  |  |
| Preoperative CEA (ng/ml) | 1.020 | 1.002 - 1.040 | 0.034* | 1.023 | 1.004 - 1.042 | 0.017* |
| Preoperative CA19-9 (ng/ml) | 1.056 | 0.977 - 1.141 | 0.167 |  |  |  |
| Preoperative biliary drainage (Yes/No) | 1.108 | 0.719 - 1.707 | 0.642 |  |  |  |
| Diagnosis (Pancreatic cancer/Others) | 1.590 | 0.926 - 2.731 | 0.092 |  |  |  |
| Surgical Procedure (PD/Others) | 0.924 | 0.632 - 1.353 | 0.686 |  |  |  |
| Operation time (hours) | 1.019 | 0.961 - 1.082 | 0.523 |  |  |  |
| Intraoperative blood loss (ml) | 1.002 | 0.976 - 1.028 | 0.878 |  |  |  |
| Blood transfusion (Yes/No) | 1.083 | 0.760 - 1.544 | 0.660 |  |  |  |
| Resection margin (R1/Others) | 1.203 | 0.588 - 2.463 | 0.613 |  |  |  |
| Lymph node metastasis (Yes/No) | 1.359 | 0.896 - 2.062 | 0.149 |  |  |  |
| Nerve invasion (Yes/No) | 21.102 | 0.043 - 10389.789 | 0.335 |  |  |  |
| Chao-yang Classification (III-IV/II) | 1.041 | 0.729 - 1.487 | 0.824 |  |  |  |
| Reconstruction procedure  (Branched AVGs/Tubular AVGs) | 0.895 | 0.627 - 1.280 | 0.545 |  |  |  |
| Postoperative complication (Yes/No) | 1.503 | 1.022 - 2.209 | 0.038* | 1.562 | 1.060 - 2.302 | 0.024* |
| Postoperative chemotherapy (Yes/No) | 0.845 | 0.596 - 1.198 | 0.345 |  |  |  |
| Postoperative vascular stenosis (Yes/No) | 1.420 | 0.962 - 2.097 | 0.078 |  |  |  |

(WBC: White blood cell; LYM: Lymphocyte; PLT: Platelet; ALB: Albumin; TB: Total bilirubin; CEA: Carcinoembryonic antigen; CA19-9: Carbohydrate antigen; PD: Pancreaticoduodenectomy; AVGs: Allogeneic vascular grafts; * *P* < 0.05)
